# Rise of remote work across borders: opportunities and implications for migrant-sending countries

**DOI:** 10.3389/fsoc.2024.1290629

**Published:** 2024-05-07

**Authors:** Inta Mieriņa, Inese Šūpule

**Affiliations:** Faculty of Social Sciences, University of Latvia, Riga, Latvia

**Keywords:** remote work, cross-border work, return migration, Latvia, Central and Eastern Europe

## Abstract

Contact restrictions imposed during the COVID-19 pandemic have contributed to the rapid expansion of remote work. With this expansion, new opportunities arise for the typical migrant-sending countries in Central and Eastern Europe to remotely involve their diaspora in their labor market. The aim of this paper is, by using the case study of Latvia, to show the potential of cross-border remote work for alleviating human capital losses caused by emigration. We assess the main obstacles and necessary adjustments in taxes, social benefits, labor market regulation and other areas to facilitate the labor market transition and show what incentives the country can use to become a place of choice for performing remote work for the diaspora. Combining the perspectives of employers, employees and the government, this study sheds new light on the challenges and opportunities related to the rise of remote work for countries suffering from emigration. The comprehensive analysis builds on triangulating secondary data, analysis of policy documents, a survey of employers, as well as a survey and in-depth interviews with cross-border remote workers.

## 1 Introduction

Contact minimization measures imposed during the COVID-19 pandemic have contributed to a rapid transition to remote work or telework at least in some sectors of economy. Although these changes are sometimes positioned as a short- or medium-term pandemic adaptation mechanism, business surveys indicate that remote work will remain popular, facilitated by the introduction and expansion of new digital technologies and tools in the recent years (Kruks et al., 2020; Okwuosa, 2020; Kniffin et al., 2021). According to European Commission research data, more than 40% of employees living in cities and 30% in rural regions currently work in professions where remote work is possible, and this is especially common for higher paid employees (almost three quarters) (European Commission, 2020). In 2020, a group of German researchers, based on a representative survey, created a teleworkability index, according to which about 31% of the workplaces could be located at home (Arntz et al., 2020).

Before the start of the COVID-19 pandemic, remote work was generally rare (MBO partners, 2019; Eurostat, 2020; Watson, 2020). For example, in Latvia just 5% of employees worked remotely in 2017 (Muižarājs, 2020)—in line with the EU average—but the number had more than tripled by the second quarter of 2020, reaching 18.3%. After culminating in the second half of 2021 at 22%, the percentage of workers performing their work remotely has now stabilized at around 11% (CSB, 2023)—more than twice the level before the pandemic.

Importantly, the pandemic accelerated changes in the work environment: The security of IT systems has improved, appropriate skills in the use of various remote working tools have been acquired, trust increased, and, finally, cultural and social barriers reduced, since both employers and employees have now experienced the benefits of remote work (Kruks et al., 2020). Among the advantages of this form of work is that the employers can save costs on renting premises, while employee satisfaction with work improves (DeRosa et al., 2007; Kniffin et al., 2021). It is expected that in the long term the number of people who work full-time in a specific workplace or country will significantly decrease, and instead we will be experiencing an increase in the so-called “digital labor migration.”

What is often overlooked in this debate, however, is that the rise of remote work increases opportunities to attract foreign labor to the labor market. Attracting workers from abroad by offering them opportunities of remote work—outside of the traditional, centralized workplace, with the help of digital technologies can, at least in some sectors, reduce labor shortages faced by many countries in Europe. If cultural threat and integration problems are the main concerns of Europeans and the reason why many are against increasing immigration (Mieriņa and Koroļeva, 2015), remote employment should be met with less resistance and generally be more successful in the long term. The structured and technologically connected international workforce can have a positive effect on innovations and productivity, as the countries benefit from the foreign professional's experience and knowledge.

Remote workers that could be easiest to attract are the diaspora, due to their established family ties, language skills, and emotional connection to the country. As diaspora surveys conducted in Latvia show, many professionals, entrepreneurs, and scientists would like to contribute their skills and knowledge to their country's development (KaŠa et al., 2021; Bela et al., 2022). The transition to remote work may also promote return migration, as diaspora members returning home often gain an opportunity to keep their well-paid employment abroad while being with their family and friends and sending children to schools in their country of origin—an advantage that is important to many (Kļave and Šūpule, 2019). To conclude, facilitating cross-border remote work has a potential to significantly benefit the country's economy and competitiveness.

Many of the Central and Eastern European countries that are typically seen as migrant-sending countries (e.g., Estonia, Czechia, Poland, Romania, the Baltic countries) are in a good position to become not only regional but also global leaders in remote work due to a fast broadband Internet, good transport connections with other European countries, a wide range of digital services, relatively fast uptake of digital technologies in the private sector, and a number of other factors (World Bank Group, 2016; Cámara, 2022; Speedtest, 2023). To take advantage of this opportunity, it is necessary to understand what incentives these countries can use to become a place of choice for performing remote work, as well as to assess more broadly the main obstacles and necessary adjustments in taxes, social benefits, labor market regulation, and other areas in order to adapt to the new conditions and to lead the process of labor market transformation.

Even though many members of the diaspora have the citizenship of the country their employer is located in, they still face various challenges and difficulties related not just to remote work as a form of employment, but also specifically to working across borders. Despite the mentioned challenges, there are few scientific publications and practical policy documents on cross-border remote employment that elaborate the main problems and the most optimal solutions related to this type of work. This is where this exploratory article intends to contribute.

Using a comprehensive methodology that triangulates policy analysis, opinions of employers and of employees, our study draws attention to the potential utility of cross-border remote work for the typical migrant-sending countries and identifies obstacles and necessary policy adjustments for them to benefit from the rise in remote work, off-setting human capital losses caused by migration.

Our study is based on an in-depth case study of Latvia as one of the countries with the highest rates of emigration in Europe. Since 2000 more than 300,000 people (13% of the population) emigrated from Latvia to another country, and there are more people with higher education among them than among the stayers (Hazans, 2020). In recent years emigration has been growing: in 2020 emigrant number reached almost 12 thousand, in 2021 almost 13 thousand, while in 2022 it accounted for 16.7 thousand, illustrating the scale of the challenge (CSB, 2023). Besides Latvia, many more countries could benefit from a better understanding of how to use the opportunities to attract employees provided by the rise in remote work.

The research questions we ask are:

1. What is the potential of cross-border remote work?
2. From the policy perspective: What are the different policy instruments and solutions in the world that are used to improve the institutional context and conditions of cross-border remote work?
3. Employers: What is the experience and attitude of Latvian employers toward cross-border remote work and the challenges it creates?
4. Employees: What is the experience and attitude of Latvian employees toward cross-border remote work and the challenges it creates?

The theory suggests that changes in the state of work caused by the COVID-19 pandemic can be best understood by looking through the lens of Pierre Bourdieu's hysteresis theory (Graham, 2020). It holds that if something moves forward but does not go back, the system has become “hysterical.” As a result of hysteresis, disturbances occur between the field and habitus, especially in cases where serious changes and crises occur in the field, and the existing order and laws are changed. Habitus falls out of the field in which it operated, loses contact with the existing order in the field, and this condition is described by Bourdieu as hysteresis (Bourdieu's, 1993). The everyday life of many companies and institutions no longer resembles what it was before. Employees may feel “disconnected” from their previous professional habits and can no longer perform their work tasks in the same way as before. On the other hand, entrepreneurs, realizing the benefits of remote work, may increasingly start using employees from countries with lower wages for intellectual labor. That is to say, the habitus of not only employees, but also employers can be expected to adapt to changes in the field.

Castells (2009) talks about people as part of a global network. Digital networks are global, they spread across institutional and territorial boundaries with the help of telecommunications and computer networks. The global network society, which operates within these networks, is also global. Using the Internet and its offered services and opportunities—wireless communications, online communication and games—a person is closely connected with other Internet users, entering the network and becoming a part of the network society. The combination of identification with a “network community” and individualism results in “networked individualism” (Castells, 2009). This concept represents a critical societal transition from geographically bounded local groups to a modern networked society consisting of permeable, sparse, and dynamic communication networks irrespective of national boundaries (Wang et al., 2018).

Some of the most discussed challenges related to remote work concern its possible effect on productivity (Schröder et al., 2020), increased difficulty of the management of human resources (GitLab, 2020; Kniffin et al., 2021), ensuring technological equipment and occupational safety of remote workplaces, as well as digital skills of workers. Cross-border work is associated with even bigger potential issues related to legislation, taxation, harmonization of social contributions, and access to basic services such as healthcare for remote workers. It also adds an extra layer of complexity to the organization of work due to the physical distance, time zone differences, the inability of workers to appear in the office at short notice, and an even weaker sense of control. From the employees' point of view, too, cross-border work could potentially aggravate previously documented problems in relation to remote work such as communication difficulties, feelings of loneliness, and the provision of adequate working conditions and workplace support outside the office (see Government of Ireland, 2019; State of Remote Work, 2020; Rācenājs, 2021). The distance may make it even more difficult to cooperate in achieving collective goals—one of the main sources of stress for workers (GitLab, 2020). In Latvia, almost half of employees working remotely consider the lack of communication and socialization to be the drawback of remote work (Kruks et al., 2020), and it is likely to be even more problematic in cross-border work situations.

Our focus in this paper is on attracting diaspora professionals as remote workers to companies in Latvia (i.e., benefiting from their knowledge and skills), even though, as mentioned before, another option—attracting return migrants who keep a job abroad could be an even more promising opportunity for countries like Latvia to benefit from the rise in remote work. For the sake of simplicity, it will be addressed in future research papers.

The structure of the paper is as follows. After discussing data and methods, we look at the potential of cross-border remote work, and then elaborate, first on the employers' attitudes, and then on the attitudes of employees toward this form of work. The article concludes with conclusions and discussion, laying the foundations for future studies in this area.

## 2 Methods and data

In this study, we define remote work as work that the employee could perform at company premises but that is constantly or regularly performed outside the company, including work performed using information and communication technologies. Cross-border remote work, accordingly, is a type of remote work where the place of residence of the worker is in a different country than the company he or she works for. Within the meaning of this definition remote work is not considered to be work that, due to its nature, involves regular movement.

To answer our research questions, we use a mixed methods approach: secondary analysis of previously collected diaspora survey data; analysis of policy documents; a survey of employers in Latvia; and a survey and in-depth interviews with members of the Latvian diaspora who either work or could potentially work remotely across borders. Quantitative surveys are key to exploring how widespread cross-border remote work is, in what type of companies and fields, and among what kind of people, and allow to acquire statistically reliable information about the attitudes of the target group. In depth interviews were important to gain a deeper understanding of the motivation, way of thinking, challenges and needs of the representatives of the Latvian diaspora working remotely. Together they provide a multifaceted complex picture of the motivations and actions of various actors involved in cross-border remote work.

To acquire information on those Latvian nationals who work remotely while residing abroad, we use data on Latvian cross-border remote employees obtained in the comprehensive online diaspora survey we conducted from September 24 to November 11, 2019 using our existing database of migrant and return migrant e-mail addresses who had previously expressed interest in participating in such surveys [further referred to as the diaspora survey] (see Mieriņa, 2019). Information about the survey was disseminated through the diaspora media (e.g., baltic-ireland.ie, latviansonline.com), placing information in Internet groups and newsgroups of Latvian citizens living abroad (16 groups draugiem.lv, three LinkedIn groups and more than 150 Facebook groups), with the help of Latvian institutions working with the diaspora (Ministry of Foreign Affairs, Ministry of Environment and Regional Development, Ministry of Education and Science, Latvian Language Agency, Latvian Investment, and Development Agency), as well as sending out information and asking to share it 390 previously identified Latvian diaspora organizations, groups, societies, congregations, etc. (including the European Latvian Association and the World Association of Free Latvians). Informative banners about the survey were placed on Facebook, on the most popular portal in Latvia DELFI, and press.lv, draugiem.lv (Latvian social media portal) invitations to its users, as well as active communication on the websites and social networks of the researchers, as well as sending of a press release. Special attention was paid to reaching Russian-speaking Latvian emigrants, taking into account that in previous studies the response rate of this group was relatively low. The achieved sample comprises 6,242 emigrants aged 15 and over. The survey includes people starting from 15 years of age, as according to the Latvian Labor Law adolescents 15–18 are allowed to be employed for up to 35 h a week (almost full-time) without requiring parental consent. For the survey data to be applicable to the entire population of Latvian emigrants, the data were statistically weighted, using multivariate data imputation, in relation to four sociodemographic factors—gender, age, level of education and main language—additionally stratifying the entire sample by the emigrants' home country. If these control variables capture most of the variation in inclusion probabilities, then the weighted data yield (approximately) unbiased and consistent estimators (Horvitz and Thompson, 1952). However, in practice these inclusion probabilities will also be affected by a series of additional factors that we were unable to correct for with survey weights (such as a respondent's intrinsic propensity to volunteer to participate in surveys). Hence, some residual deviations from full representativeness will remain. However, these deviations are likely to be minor, of an order of magnitude similar to the deviations that non-response would cause in a simple random sample. Data from the OECD, Eurostat, the Citizenship and Migration Affairs Office of the Republic of Latvia and the Office for National Statistics of the UK were used as the basis for information on the general population of Latvian migrants (more in Goldmanis and Mieriņa, 2021). To identify the target groups, an affirmative answer about the main job as “remote work in a company or organization in another country” was used. Of the total number of respondents, 162 (3.8%) performed remote work for a company or organization in another country among the interviewed representatives of the diaspora.

To obtain information about employers' experience with and attitude toward remote work, a representative survey of Latvian employers was conducted from 26 of February to 25 of March 2021. The selection of respondents was carried out by applying multistage quota sampling, with companies divided into sectors according to NACE codes, i.e., the size of quotas was determined in proportion to the sector's contribution to the creation of Latvia's gross domestic product. As our intension was to focus on private companies operating in Latvia (recruitment practices in the government sector are determined centrally), the survey does not include companies and institutions operating in: (O) state administration and defense, and compulsory social insurance; (T) activities of households as employers, and production of self-consumption goods; and (U) activities of extraterritorial organizations and institutions. The achieved sample size was 750 respondents.

To gain a deeper understanding of the motivation, way of thinking, and needs of members of the Latvian diaspora who work remotely in another country, 11 semi-structured in-depth interviews with representatives of this group were conducted. The average length of interviews was 48 min, and they took place from June 7 to August 3, 2021. Interviews were conducted remotely using different communication platforms, depending on the interviewee's preference (Skype, Zoom, Webex, Google Meet, or Microsoft Teams). Before the interview, communication with the interviewees took place by e-mail, e-mail was used both to obtain informed consent from the interviewees for participating in the study, and to agree on the time and the most convenient format of the interview. Before the recording was made, the interviewee was again informed that the interview was being audio recorded. The audio files were transcribed, and thematic coding was carried out. The process of recruitment revealed that migration patterns are very fluid, and people's situations–variable. Several interviewees were engaged in circular migration, spending at least some months of the year in Latvia. Among the interviewees were 6 women and 5 men aged 25–55. Their countries of emigration are Germany, Sweden, Greece, Spain, Belgium, Bulgaria, and others. The interviewees work in programming, tourism, translation, research, communication, business analytics, sales, and other fields. Almost all have long-term remote work experience (complete interviewee characteristics in Table 1). The interviews have been anonymized to protect the identity of the research participants. After preparing the transcripts, the audio files were deleted, while the transcripts were prepared in a format that excludes the possibility of identifying the person who gave the interview (the name of the transcript file has been coded, the name of the interviewee and other very personal information, for example, about the address of residence, if mentioned in the interview, has been deleted).

**Table 1:** Interviewee characteristics.

**RN**	**Country**	**Profession/field**	**Status**	**Gender**	**Education**
1	Greece	Economist, NGO projects	Emigrant	F	Doctoral degree
2	Malta	Marketing	Emigrant	M	Secondary education
3	Spain	Translator	Emigrant	F	Bachelor's degree
4	Russia	Engineer	Emigrant	M	Bachelor's degree
5	Germany	Communications designer	Emigrant	F	Bachelor's degree
6	Belgium	Sales expert	Emigrant	F	Masters degree
7	Spain	Tourism	Circular migrant	M	Bachelor's degree
8	Germany	Commercial business analytics	Circular migrant	F	Masters degree
9	Bulgaria	Musician, Crypto-analytic	Circular migrant	M	Secondary education
10	Sweden	Programmer	Circular migrant	M	Bachelor's degree
11	Sweden	Analyst, researcher	Transnational migrant	F	Doctoral degree

Finally, a quantitative survey of remote workers was carried out from September 10 to September 24, 2021 [further referred to as the survey of remote workers]. Unlike the diaspora survey, it allowed statistically reliable information about the situation and attitudes of the target group to be obtained. Like in the diaspora survey, to recruit respondents, researchers used the unique database of e-mail addresses of Latvians living abroad who have agreed to participate in further research by the University of Latvia. To expand the circle of respondents, recruitment was also carried out on the websites and social networks of the organizations that work closest with the diaspora (Ministry of Foreign Affairs, Investment and Development Agency of Latvia, diaspora NGOs), as well as with targeted Facebook ads. The following people were asked to participate in the survey:

1. those members of the diaspora who currently work remotely or from home (regardless of where their employer is based) or,
2. those for whom the specifics of their employment would allow the work to be carried out fully or partially remotely or without attachment to a specific place.

In total, 559 remote workers who currently live abroad (in 54 countries) participated in the survey. The diaspora survey and the survey of remote employees, each having a different source of funding, nicely complement each other—one with substantially larger sample size, allowing to acquire information on how widespread cross-border remote work is, and the other with more in-depth information on the specific target groups.

In collecting, processing and analyzing the data, all EU-level and nation-specific legal regulations regarding ethics, confidentiality, and data protection were strictly followed. In all cases, voluntary informed consent was sought from research participants. In line with EU Directive 95/46/EC, respondents were informed as to how their data will be processed, stored, and identity protected. All efforts were made to ensure participants fully understand the implications of being involved in the research, including conveying the information in the language they understand best. This information was conveyed to respondents in written form (surveys) or oral form (interviews) before the start of the interview, and the consent was also provided in either written or oral form. Clearance from the Ethics Committee for Humanities and Social Sciences at the University of Latvia was obtained for both qualitative and quantitative parts of the study. The employer's survey was conducted by an external experienced research company SKDS who are bound by their own strict ethics rules and practices.

## 3 Results

### 3.1 Regulations concerning remote workers

European Union guidelines “Frontier Workers in the European Union” contain some regulations on cross-border work and information on the rights of cross-border workers (European Parliament, 2021). As the first fundamental problem, the report acknowledges defining remote work. There are different possible variants of the employee's involvement in the activities of the home country and the time spent in the home country, and the bilateral agreements of the countries on the tax policy of the employee and the employer include different definitions of cross-border work. Regarding the rights of cross-border workers within the EU, the document states that in accordance with the principle of equal treatment and the right to freedom of movement, they have the same rights as local residents in the country where they work (in line with EEC Regulation No 1612/68, Article 7).

However, in practice, it is not so simple. The rate at which the income of a cross-border employee is taxed depends on bilateral taxation agreements, signed with the aim of avoiding double taxation of trans-national income. The rules and criteria stipulated in these agreements also differ—there are cases when the income of a cross-border employee is taxed in the home country (e.g., the Franco–Belgian double taxation agreement) or in the country where the employer is based at (e.g., the agreement between the Netherlands and Germany), or in both countries at the same time (the agreement between Switzerland and Germany). Without going into too much detail, national agreements are very different, and each country's tax system and conditions differ significantly. Tax compliance issues are so complex that employers willing to attract cross-border workers often hire global financial advisory companies or do a thorough study of tax systems to understand what hiring such workers would entail and what to expect.

The guidelines of the European Union on cross-border work “Frontier Workers in the European Union” (European Parliament, 2021) indicate the following problem aspects for cross-border work: (1) social security—receiving various benefits (disability benefit, pension, etc.), social guarantees, access to health care, (2) taxation; (3) relations between both countries—differences in the amount of taxes and social contributions, mandatory level of contributions; (4) use of a work vehicle in the country of residence and the country of employment; (5) lack of information, problems with cooperation between the two participating countries.

The report published by Deloitte (2021) on the problems of remote work across borders offers an even more detailed insight into the problems and uncertainties facing companies employing cross-border remote workers: (1) filing annual individual tax return; (2) payroll reporting and employer filing/reporting; (3) immigration considerations/right to work; (4) employment law compliance and regulatory compliance; (5) permanent residence/tax residence considerations; (6) employee transfer pricing; (7) intellectual property location and corporate structure; and (8) indirect tax and withholding tax exposure. State-level cooperation in facilitating and implementing remote work has been highlighted as important in earlier reports (Government of Ireland, 2019).

Cross-border workers encounter even more obstacles than their employer, including those that can prevent them from staying in a place for a longer period of time—for example, it is not possible to apply for a work visa because the job is not located in the country where they plan to stay, but the tourist visa is not valid for long enough, and it is not intended for work. Our diaspora survey reveals that 18% of the members of Latvian diaspora who work remotely are not citizens of Latvia, and more than a third have a partner who is not a citizen of Latvia. This can make it difficult for them to move to Latvia, if such a decision were made.

To rectify these issues, more and more countries are introducing digital nomad visas (ETIAS, 2021). For example, in Estonia, remote workers who do not have Estonian citizenship can apply for a digital nomad visa, which allows them to live and work legally for employers in Estonia or in their own company registered abroad for up to a year. In this way, uncertainty about the immigration status of telecommuters, who often work remotely in the country on tourist visas, is reduced. Persons without Estonian citizenship can also apply for e-residency, which allows them to receive a state-issued digital identity card enabling electronic authentication and receiving electronic public services. Among other things, it allows individuals to remotely register companies in Estonia, submit tax returns and gain access to the Estonian banking system. In addition to Estonia, currently, the digital nomad visa can be issued in the following European countries: Croatia, Czech Republic, Germany, Greece, Iceland, Italy, Portugal, Norway, and (since 2022) also in Latvia, and in the following countries outside Europe: Antigua & Barbuda, Barbados, Bermuda, Costa Rica, Cayman Islands, UAE, Georgia, Australia, Mexico, Mauritius, Thailand, and Curacao. Around 35 million people are employed worldwide using the digital nomad visa. Conditions for granting such a visa vary, but most often they are a valid passport and proof of income. In Latvia, the digital nomad visa gives citizens of third countries the right of residence in Latvia. The right to employment in Latvia is not granted to a digital nomad. Digital nomad visas make it easier for those members of the diaspora who do not have Latvian citizenship, as well as their unmarried partners, to move to Latvia while keeping their job abroad, but they do not help those wishing to work in Latvian companies while residing abroad.

The great human capital in this group of employees and the usefulness of attracting them to the labor market is illustrated by the fact that cross-border remote work is much more typical of the better-educated representatives of the diaspora: According to our diaspora survey, 64% have a higher education, and half of them obtained at least one level of education abroad. Moreover, those working remotely significantly more often than other working members of the diaspora admit that they could, depending on circumstances, return to Latvia within the next 5 years.

### 3.2 The current state of remote work and cross-border employment

Opportunities to work remotely depend to a large extent on the sector of employment, but certain limitations may also stem from the geographical proximity of the workplace. For those wishing to work remotely from another country, only those positions that allow full-time or close to full-time remote work are suitable. The global survey “State of Remote Work” conducted in 2020 revealed that 57% of remote employees worked remotely 100% of their working time, and another 16% did so for 76%−99% of their working time (State of Remote Work, 2020). Our own survey of remote workers closely aligns with these numbers: 62% of diaspora members work remotely for more than 75% of their working time. Those who work remotely only partially were asked whether the specifics of their job would allow them to work completely or almost completely remotely. The answers show that it would be possible in more than half of the cases. It must also be noted that living in another country does not necessarily make partial remote work impossible—as our survey shows, 30% of cross-border workers work remotely part-time and spend at least some time at the office, while 22% admit that they alternate between Latvia and another country (i.e., have a transnational way of life).

**Figure 1:**
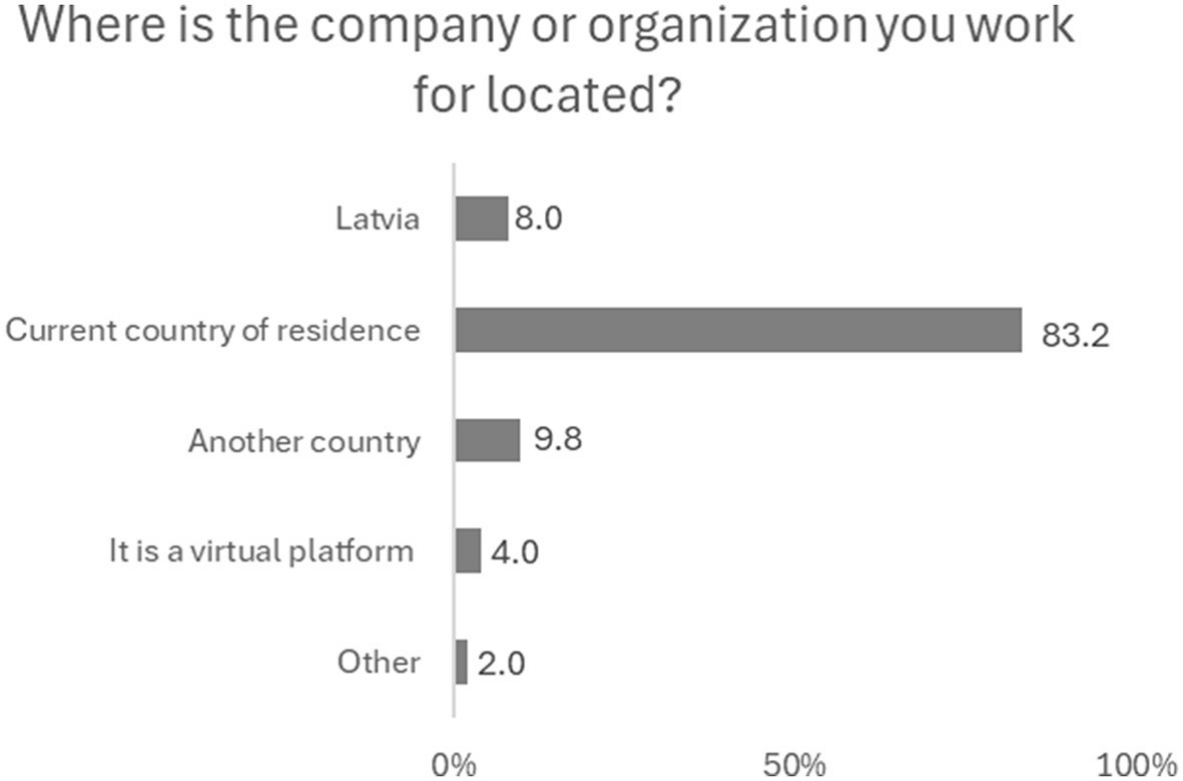
In which country are the remote workers employed? (%). Source: survey of remote workers (2021).

Considering that most remote workers currently work fully or almost fully remotely, they could potentially work in a country other than their country of residence. Nevertheless, members of the Latvian diaspora who currently work remotely while living abroad, are most likely to be employed in their country of residence (83.2%). Importantly, just 8% of remotely employed members of the diaspora work in companies or institutions in Latvia. In fact, it is more common for them to work remotely for a company in another foreign country than in Latvia (Figure 1). This shows that only a few of those specialists who can and do perform their duties remotely across borders choose to invest their knowledge in Latvian companies and institutions.

### 3.3 Attitudes of employers

The employers' survey in Latvia conducted in 2021 reveals that in half of companies (51%) there are at least some employees who work remotely, including in almost all companies providing information and communication services. In companies that are, in principle, open to remote work, a large proportion of employees work remotely. For example, 58% of companies in the IT industry have all or almost all employees working remotely. About half of companies operating in finance and insurance, professional, scientific, and technical services, and in education also have nearly all or all employees working remotely. The diaspora survey reveals that employees working in IT make up a third (32%) of all representatives of the Latvian diaspora and return migrants working remotely in another country; however, as we can see, there are many areas with possibilities to attract remote workers from abroad.

Nevertheless, the practice of employing employees who live permanently abroad is currently very rare in Latvian companies: such employees can be found in only 4.4% of companies that practice remote work, and most of them have foreign capital.

**Figure 2:**
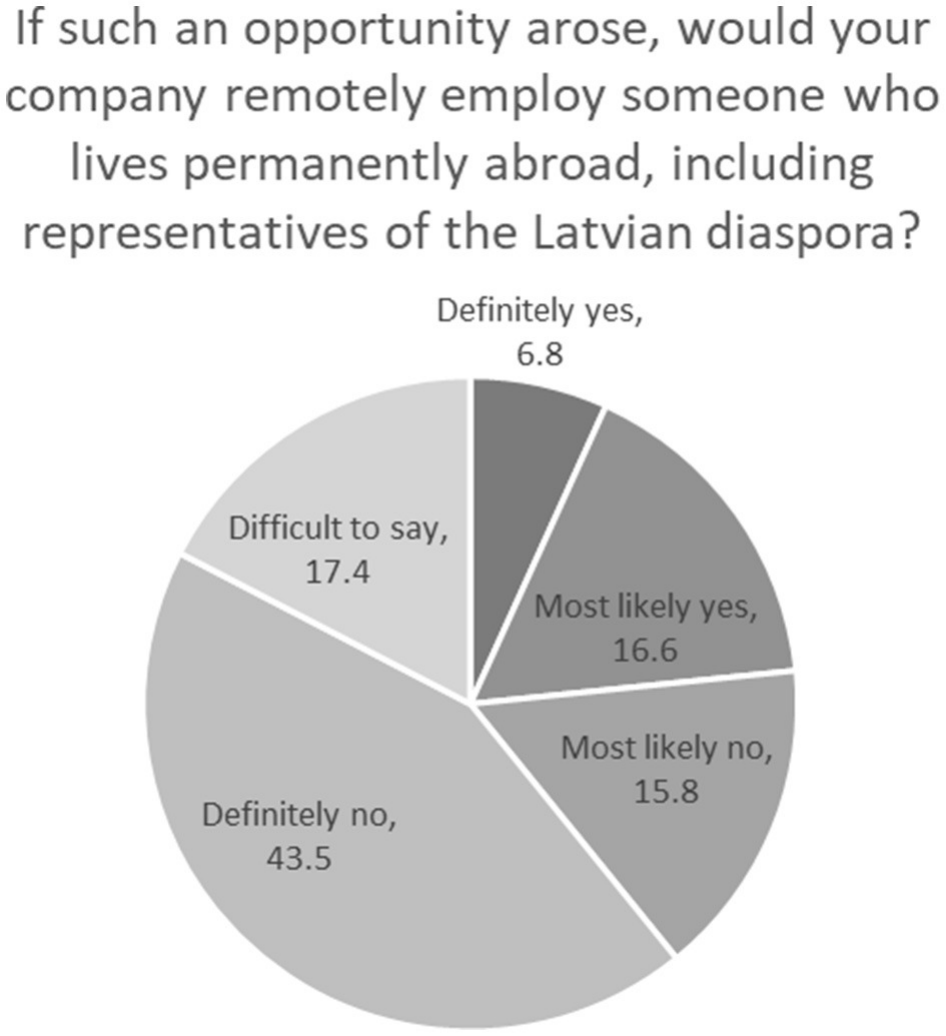
Employers' attitude toward employing cross-border workers (%). Source: survey of employers (2021).

The surveyed companies that currently do not employ cross-border employees were asked about their attitude toward the possibility of remotely employing (more) employees permanently living abroad, including representatives of the Latvian diaspora. The answers provided reveal a great potential of this form of work: If currently only 4.4% of companies practicing remote work have remote employees abroad, in the future 6.8% would certainly use an opportunity to employ such workers, and 16.6% would most likely use such an opportunity (Figure 2). The IT sector is relatively the most open to remote employment (37%), probably due to the specifics of work and the shortage of employees in the sector. At the same time, the majority of companies have a strongly negative attitude, which is probably related to perceptions of the difficulties that could arise in this type of employment relationship.

It is interesting that the attitudes toward this type of employment in companies that already have remote workers living abroad are not clear-cut. Approximately half of them most likely or definitely would not like to remotely employ more employees who live permanently abroad, but just less than a third would use such an opportunity. The data show that more often than other companies who employ remote workers, they have faced difficulties with the lack of digital skills of their remote workers, as well as with the control and registration of their working hours (even though to the small size of the group the difference is only significant at 0.1 level).

To reveal what may drive the rejection of cross-border remote employment, we ran a multinominal regression of the difficulties that the companies employing remote workers face on their attitudes toward cross-border work. Our reference category is “Would employ” (consisting of a combined “Definitely yes” and “Most likely yes”), as we are also interested in contrasting it with “Difficult to say.” We also include as control variables the size of the company, how many employees currently work remotely, how big is the role of digital technologies in the company, what capital is represented in the company (local or foreign), as well as the sector of economic activity. They all could potentially affect whether the company would or would not be ready to expand their pool of employees from across the borders (or to allow their current remote employees to work from abroad).

The overall fit of the model is very good—it explains attitudes toward employing cross-border employees significantly better than the empty model (Sig. = 0.005), and based on the Nagelkerke R2 suggests that, overall, 22% of variation in responses is explained by the specified model (Table 2).

**Table 2:** Multinomial regression of difficulties the company faces on willingness to employ cross-border remote workers (with controls).

**Ref.cat. Would employ**	**Would not employ**				**Difficult to say**			
	**B**	**S.E**.	**Sig**.	**Exp(B)**	**B**	**S.E**.	**Sig**.	**Exp(B)**
Intercept	2.390	0.871	0.006		1.419	1.071	0.185	
**Difficulties the company faces**								
Control and registration of working hours of remote workers	0.263	0.461	0.567	1.301	−0.640	0.688	0.352	0.528
Providing adequate working environment outside the office	−0.120	0.434	0.783	0.887	0.255	0.559	0.648	1.291
Providing employees with technology and resources outside the office	−0.298	0.440	0.498	0.742	−0.395	0.578	0.494	0.673
Insufficient digital skills of employees	−0.256	0.481	0.595	0.774	−0.717	0.751	0.340	0.488
Difficulties in arranging signatures or other formalities	0.461	0.432	0.286	1.586	−0.593	0.676	0.380	0.552
Less efficient work organization	0.112	0.425	0.791	1.119	0.244	0.575	0.671	1.277
Difficulty of separating private and work life	1.042^*^	0.421	0.013	2.834	0.545	0.542	0.315	1.724
Communication outside regular working hours	0.150	0.464	0.746	1.162	−0.639	0.660	0.333	0.528
Difficulty to maintain team spirit	−1.474^**^	0.444	0.001	0.229	−0.793	0.566	0.161	0.452
More frequent misunderstandings or confusion	0.877~	0.493	0.075	2.404	−0.073	0.702	0.917	0.930
A slower process of communication	0.212	0.413	0.608	1.236	0.486	0.549	0.375	1.626
No difficulties	0.598	0.425	0.160	1.818	0.210	0.549	0.701	1.234
Hard to say	0.565	0.930	0.543	1.760	1.043	0.994	0.294	2.839
Digital technologies have an important role in the company	−0.877^*^	0.401	0.029	0.416	−1.079^*^	0.464	0.020	0.340
**How many employees work remotely (ref.cat.: Less than half)**								
Half or slightly more than half of employees work remotely	−0.944^*^	0.433	0.029	0.389	−1.129^*^	0.562	0.045	0.323
All or almost all employees work remotely	−1.321^**^	0.469	0.005	0.267	−0.750	0.571	0.189	0.472
**Sector of economic activity (ref.cat.: Manufacturing)**								
Services	−0.914~	0.502	0.069	0.401	−0.160	0.660	0.808	0.852
Trade	−0.767	0.630	0.223	0.464	0.665	0.768	0.387	1.944
Construction	−1.780^*^	0.765	0.020	0.169	−0.828	0.981	0.399	0.437

Base: at least one employee works mostly or entirely from home and there are currently no remotely employed employees living abroad. Source: The survey of employers (2021). Note: ^***^Effect significant at < 0.001 level; ^**^ − < 0.01 level; ^*^ = 0.5 level, ~ − 0.1 level. The number of employees and the capital represented did not have a significant effect, so they are not presented in the table.

The analysis shows that companies that are significantly less likely to use the opportunity to employ someone who lives permanently abroad, including representatives of the Latvian diaspora, are worried about remote workers' difficulties to separate private and work life, and about the frequent misunderstandings and confusion caused by remote employment. The effect, even though in the latter case it is only significant at 0.1 level, is quite large: the companies where these concerns are present are 2.4–2.8 times more likely to avoid cross-border employment. Interestingly, those that are struggling with maintaining team spirit show more interest in employing someone who permanently lives abroad. It is possible that by attracting foreign employees they expect to boost team morale.

Some other interesting findings can be drawn from the data. For example, companies where digital technologies play an important role, are significantly more open to cross-border employment; they may have more confidence in their abilities to solve any potential technological issues related to cross-border work (e.g., data transfer and security). Openness to cross-border employment also depends on the number of remote workers currently employed by the company: companies where all or practically all employees work remotely are almost four times more likely to be interested in employing someone who permanently lives abroad than those in which less than half of employees work remotely (Table 2). Finally, companies working in the service sector or construction would be more interested in employing remotely someone from abroad, whereas manufacturing companies would be much less likely to be interested in such an opportunity.

If we look at differences between those companies who would be interested in employing someone from abroad, and those who found it “Difficult to say,” we see that hesitation is often typical to those companies that are not so technologically advanced, and where a relatively small number of employees work remotely.

When asked directly, in an open-ended question, to name any obstacles preventing the company from employing (more) employees who live permanently abroad, the most frequently mentioned answers—each mentioned by more than a third of respondents—are that the specifics of the job do not allow such employees to be employed (e.g., most employees need direct contact with customers, need to be on-site, undergo regular training, etc.) or that foreign labor is not of interest, it is not needed, either because more workers are not needed in principle, or they can be found locally in Latvia. Some employers are worried about possible communication problems, as many consider it necessary to meet in person at least now and then. Legal difficulties related to both the employment of such employees and the processing of documents are also often mentioned, including making sure that the workplace is equipped according to the regulations. Some are worried about the tax issues, about which there is a lack of clarity. Others mention concerns about the adequacy of the qualifications of such employees. Still others are worried about how it is possible to carry out the selection and recruitment of employees abroad. Data security issues are also mentioned among the problems, as well as the fact that such employees are more difficult to control. As one of our in-depth interview participants notes:

*I think this option is largely based on the manager's opinion. She sees what you do, how you work, and she already knows you. [..] If there are people she can rely on, then she doesn't need them directly in the office*. (RN1_2021)

Moreover, as the interviews suggest, this also depends on the worker being valuable enough for the company to take on the technical and legal difficulties related to cross-border employment.

While the survey of employers does not include the government sector, the diaspora survey reveals that mostly members of the Latvian diaspora who work for an employer in another country are employed by private companies (64%) or international organizations (23%), and just 3%—at a state or municipal establishment. This confirms earlier findings of other studies (Government of Ireland, 2019) that opportunities provided by the rise of remote work so far have not been utilized to the same extent in the public sector.

### 3.4 Difficulties and benefits related to remote work: employee perspective

**Figure 3:**
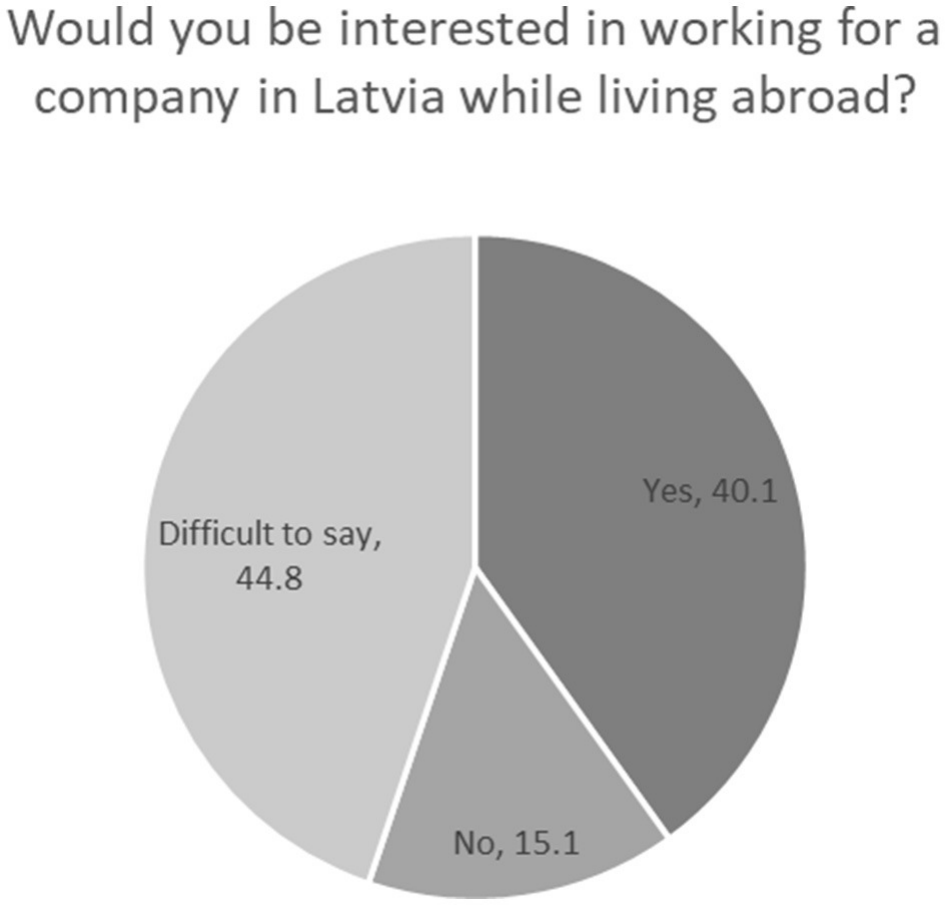
Attitude toward cross-border remote work (%). Source: survey of remote workers (2021).

To estimate the potential of cross-border remote work, we asked our respondents who currently work remotely in the same country they live in, whether they would consider cross-border employment. The answers show that of those who currently live and work remotely abroad, 40.1% would be ready to work remotely for a company or organization in Latvia (while living abroad), while many found this question difficult to answer, as it depends on various circumstances and conditions (Figure 3). The number of remote workers outright rejecting the idea of cross-border work is relatively small.

Our diaspora survey shows that those who already work across borders are very satisfied with their job (8.2 on a scale from 0 to 10, Table 3), and 77.6% would like to continue working like this in the future (another 20.7% found it difficult to formulate their attitude). They are also as satisfied as other Latvian emigrants with their family life, relationships with other people, housing, and the overall living conditions. Companies in Latvia have mostly been able to offer competitive wages to representatives of the diaspora working remotely: 43.6% of employees living abroad and working for companies in Latvia rate their salary compared to the salary of specialists in a similar field in their country or residence, as approximately the same, 24%—as higher, and only 20% believe that it is lower. Overall, this confirms the high appeal and potential of this form of employment from the employee perspective. At the same time, 44% of those members of the Latvian diaspora who currently live and work remotely in another country, expressed willingness to return to Latvia as soon as an opportunity arises.

**Table 3:** Satisfaction with various aspects of life (0–10).

	**Diaspora**	
	**Remote job abroad**	**Other kind of job**
Main job	8.2	8.2
Work conditions	8.2	8.4
Salary	7.8	8.0
Family life	8.1	8.2
Relationships with people outside family	8.0	8.2
Housing	8.0	8.0
Current standard of living	7.8	8.0
Life in general	7.9	8.2

Source: survey of Latvian diaspora (2019).

Both our qualitative and quantitative data suggest that at least some of the workers deliberately looked for opportunities to work remotely in a country other than where they live (or, on the contrary, to live in a country where their workplace is not located). Some are fascinated by the freedom of movement it provides and had a long-term strategic goal “*to do everything technically possible so that I can work from anywhere in the world*” (RN3_2021). For others, remote work is a solution in the case of cross-border relationships that makes a transnational way of life possible: “*With my remote work, I can negotiate anything. I can arrange for a couple of months a year when I'm on the other side of the world.”* (RN11_2021)

**Figure 4:**
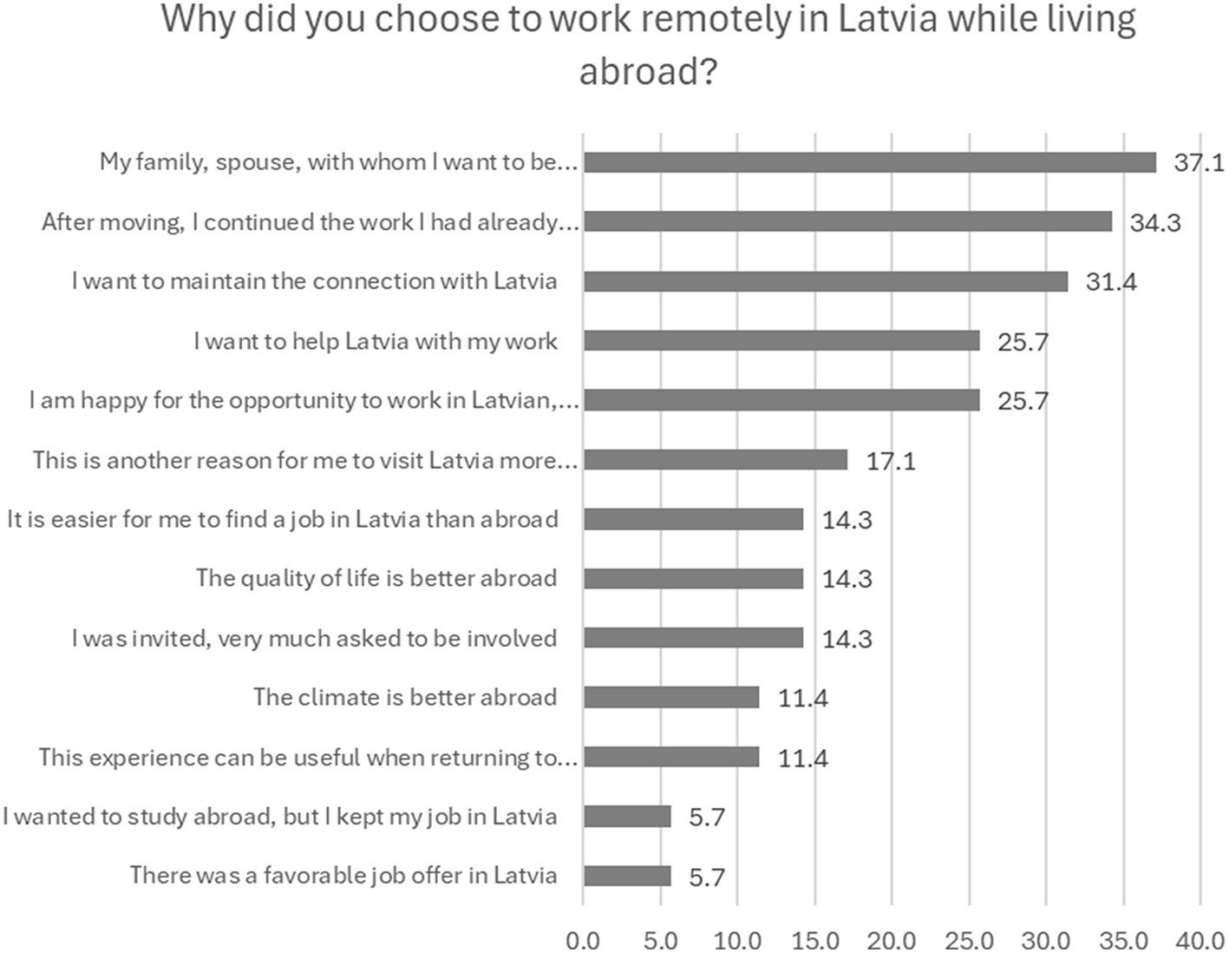
Why did people choose to work remotely in Latvia while living abroad? Source: survey of remote workers (2021).

The interviews reveal that cross-border work, particularly a job in their home country, is also especially sought by expats who have poor knowledge of the local language and therefore find it difficult getting a job in their new country of residence. Often these are spouses who have joined their husband or wife abroad. According to our quantitative survey, a total of 37% of those who live abroad but work remotely in Latvia mention that their family, spouse, children live outside of Latvia and therefore they cannot move to Latvia, even though they would like to and it would be more convenient for them. Of these, 34.3% have their remote job in Latvia because they wanted and had an opportunity to continue this work even after moving to another country. The final most important group of motives is a set of psychological-emotional factors. For many of those living abroad, the possibility of working remotely in Latvia is related to the desire to maintain or at least not lose the connection with Latvia: 31.4% directly admit that they want to maintain the connection with Latvia in this way; while 25.7% want to help Latvia with their work (Figure 4).

17.1% appreciate that work in Latvia serves as an additional reason for them to visit Latvia more often. A fourth of the respondents (25.7%) are happy about the opportunity to work in Latvian, with Latvian colleagues, while 11.4% indicate that this work experience can be useful when making a decision about a possible return to Latvia. However, other facts pointed out by some respondents are also quite important for choosing to work in Latvia while residing abroad—the quality of life abroad is higher (14.3%) and also the climate is better (11.4%). Similarly, 5.7% of respondents went to study abroad, but did not want to stop working in Latvia, so they continue to work remotely.

**Figure 5:**
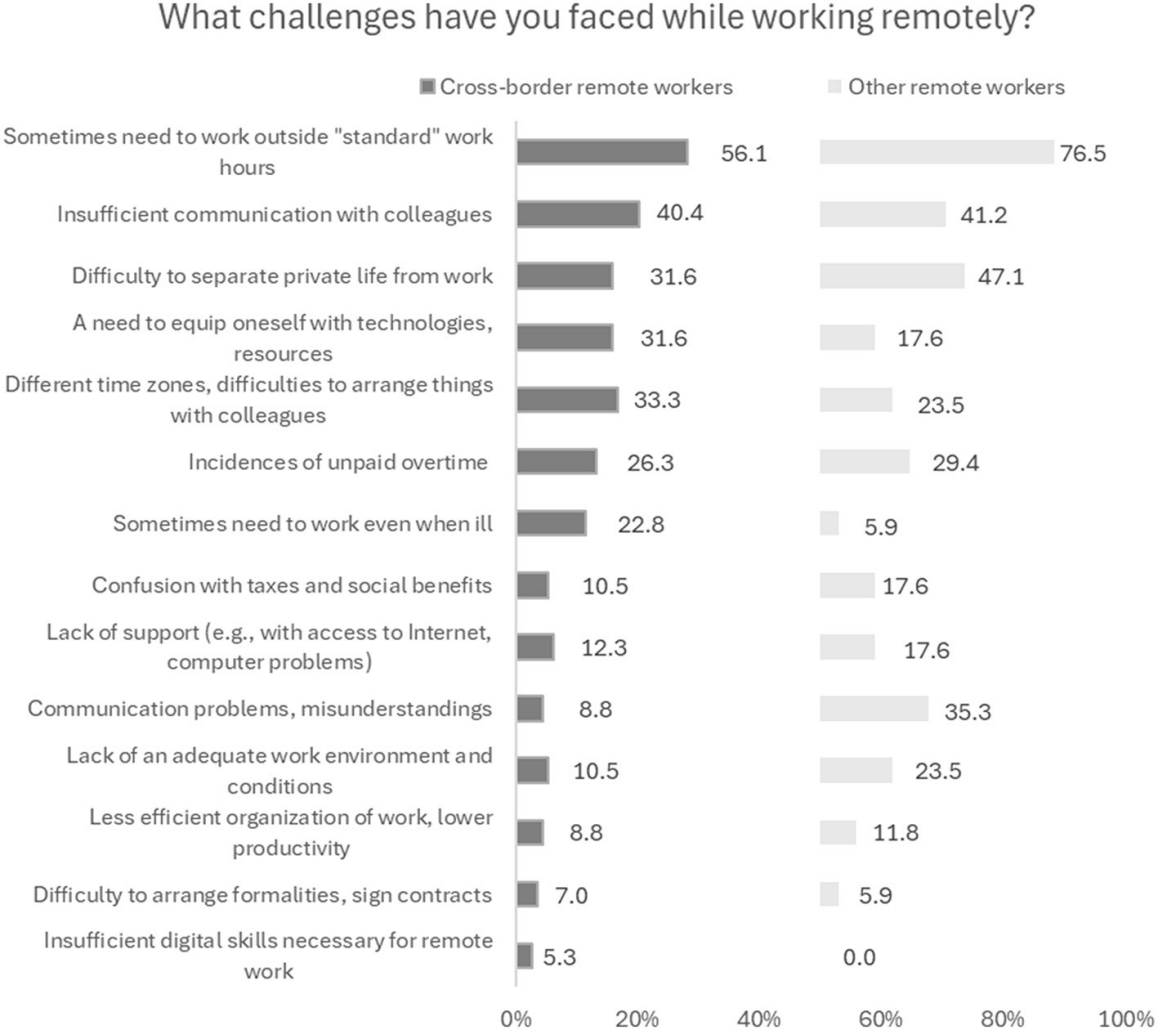
Challenges while working remotely (%). Source: survey of remote workers (2021).

Next, we turn to the analysis of difficulties related to cross-border remote work. Just as for other remote workers, some of the biggest problems for these workers is that they sometimes have to work outside “standard” working hours (56.1%) and that they have difficulties in separating private life from work (31.6%). Cross-border remote workers also complain about insufficient communication with colleagues (40.4%) (Figure 5). The sense of great isolation both in relation to the company they work in and in relation to their work colleagues also transpires in in-depth interviews. Nevertheless, the interviewees emphasize that this is not an unsolvable problem, and everything depends on the employer's ability to improve the communication flow. Interestingly, in cross-border work situation communication problems and misunderstandings seem to be much less frequent (Figure 5)—probably because the colleagues are often culturally closer (in case remote work is in a company in their country of origin) or much of the communication is in a written form and easy to verify.

Compared to those working remotely in their country of residence, cross-border remote work more often requires equipping oneself with the necessary technologies and resources (31.6% vs. 17.6%). Even though the law requires employers in Latvia to cover the remote employee's expenses related to the remote work, and even envisages a small tax credit for this purpose (30 euros per month), none of our interviewees had had these expenses reimbursed in full by the employer. Nevertheless, cross-border employees manage to deal with this problem: just 10.5% compared to 23.5% among other remote workers complain of a lack of adequate work environment and conditions.

Interestingly, our survey finds that cross-border workers more often have had to work while ill (22.8%). This might be related to the difficulties in arranging the formalities for sick leave in cases where the person falls ill in a country other than their workplace. Another reason is that sometimes cross-border workers avoid going to the doctor as much as possible as they are not sure whether the expenses will be covered by the social security. As our survey shows, at least one in ten cross-border remote workers, particularly those who work for a company in another country, not Latvia or their current country of residence, do not know where they qualify for healthcare services or think they cannot get them anywhere (Table 4).

**Table 4:** Social services and taxation for cross-border workers in the diaspora (%).

**Where..**.	**In Latvia**		**I another country**		**Difficult to say/Don't know**		**Nowhere**		**Don't want to answer**	
	**Works for a company in Latvia**	**Works for a company in another country**	**Works for a company in Latvia**	**Works for a company in another country**	**Works for a company in Latvia**	**Works for a company in another country**	**Works for a company in Latvia**	**Works for a company in another country**	**Works for a company in Latvia**	**Works for a company in another country**
… taxes are paid for your remote work?	68	15	18	75	5	0	5	6	5	8
… you are registered as a tax resident?	67	23	38	70	0	0	0	4	5	8
…you can access public health care?	55	25	55	75	5	8	0	8	5	4
…you can claim maternity, paternity, parental, childcare benefits?	45	13	35	70	20	9	10	8	5	4
… you can claim unemployment benefit?	33	10	29	63	19	10	19	15	5	4
… contributions are made to your pension from your remote work?	55	15	32	70	14	2	9	11	5	6

Source: survey of remote workers (2021).

Cross-border remote workers sometimes work in another time zone, making it difficult to arrange things with colleagues (33.3%, Figure 5), and their colleagues suffer from this too. Some have found it difficult to complete various formalities or sign contracts in a cross-border employment situation; however, this number is small (7%). Confusion with taxes and social benefits is sometimes a challenge (10.5% of those working from abroad in Latvia), however, this is less of a problem for them than for the other remote workers—probably because if their country of employment is Latvia, they are more familiar with its tax system than that of another country.

When asked directly, more than a third (35%) of the representatives of the diaspora working remotely across borders admitted that they had encountered difficulties or lack of clarity in tax matters, and 13% had a lack of clarity or problems around social benefits. The three main issues, according to our respondents, are: lack of clarity with the application of taxes in specific cases; a general complexity of tax systems and lack of information/difficulty navigating this information; as well as bureaucracy and a lack of understanding on the part of state institutions.

Respondents' answers reveal that the majority (about two thirds) of cross-border remote workers are registered as tax residents and pay taxes in the country they live. However, two thirds of those who work for a company in Latvia, are registered as tax residents and pay their taxes in Latvia (Table 4). According to the Latvian tax laws, with some exceptions, a person will be considered a resident if: this person's declared place of residence is in Latvia or this person stays in Latvia for 183 days or more in any 12-month period. Thus, one can assume that for various reasons (lack of information on tax matters, plans to possibly return, transnational lifestyle) many members of the diaspora working remotely for a company in Latvia have not formally moved their tax residence to where they currently live, as they should have. Twenty three percentage members of the diaspora working remotely in another country are still tax residents in Latvia, and some are tax residents in Latvia and in another country simultaneously. 28.8% of cross-border workers living abroad do not know where they can claim unemployment benefits, and 20.8%—maternity/parental benefits or think that they cannot get them anywhere. The lack of clarity regarding eligibility for social benefits is especially widespread among those members of diaspora who work in Latvia (Table 4). For the tax residents of other countries, it is possible to voluntarily join the state social insurance system in Latvia; however, the majority of the respondents do not know about such an opportunity, and so far, a very small number of respondents (3.8%) have used it.

Uncertainty about how to legally formalize remote work, and how to organize tax payment, is one of the factors that discourages cross-border remote work.

*It is not clear. Where will I pay those taxes? Who will pay? How will it be paid? In Latvia or abroad? Because you already must come to the employer with an offer of how it could work. And people don't know those solutions. There is no clear, convenient way to do it*. (RN10_2021)

The interviewees conclude that those who work remotely across borders need specific personal characteristics that are more typical of entrepreneurs than simple workers: First, they need to be able to work independently, organize their time to cope with the task, respecting the deadlines, to be disciplined. Second, they also need to be able to arrange, both practically and legally, the possibility of working remotely across borders. Cross-border workers also find it more difficult to secure bank loans which require proof of income.

Self-employment is a common choice of cross-border workers. This choice is associated with greater responsibility for paying one's own taxes, and therefore also social security, as well as accounting for one's expenses and reporting to the State Revenue Service. Another legal avenue to formalize remote employees abroad is for the company to find an intermediary organization that officially employs these workers and pays the taxes in this country. Such a model is sometimes more acceptable to the company, as all the requirements stipulated in the regulatory framework have been met; however, such a cooperation model is also more expensive for the employer. The first investment is to find out the regulations of the other country which results in consultants' expenses. Additional expenses come from hiring an intermediary organization (accounting expenses). Interviewees also note that when working remotely for a Latvian company or organization from anywhere in the world, so far it has not been clearly defined in regulations how the physical workplace of the remote worker should be handled and fixed in the contract, thus, in some cases, as our data shows, a person simply continues to work as a resident of Latvia despite physically having moved to another country.

**Figure 6:**
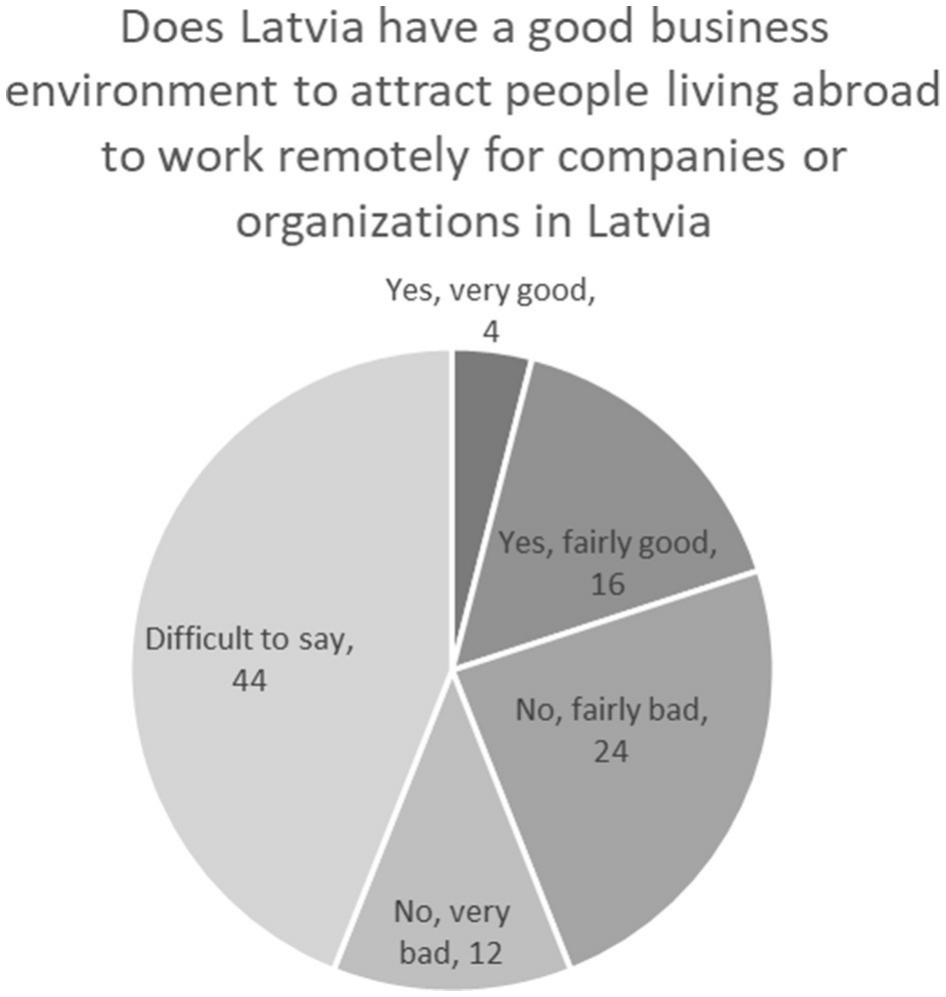
Perception of the competitiveness of Latvia as a place for remote employment (%). Source: survey of remote workers (2021).

Talking about the potential of remote cross-border work in Latvia (Figure 6), the diaspora representatives' assessment of Latvia as a business environment to attract people living abroad is not very optimistic. Only 20.3% of respondents rate Latvia's business environment as good for attracting people living abroad to work remotely for companies or organizations in Latvia. Instead, many assess Latvia's business environment as “quite bad” (23.9%) or “very bad” (12.3%). A large number of respondents (43.5%) refrain from assessing the business environment in Latvia as they are not sufficiently familiar with it (Figure 6).

**Figure 7:**
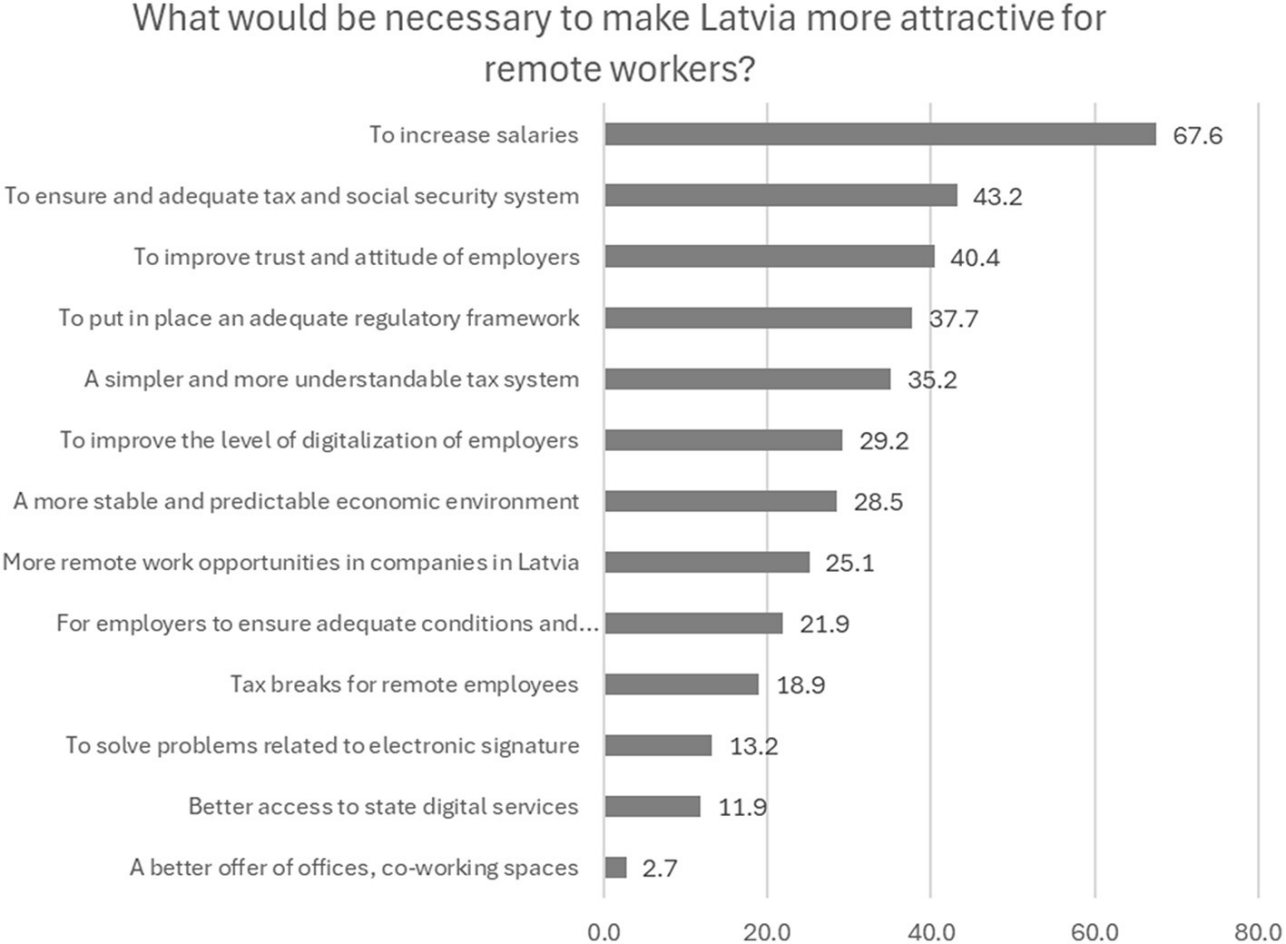
What improvements would be necessary to make Latvia a more attractive place for remote workers? (%). Source: survey of remote workers (2021).

When answering the question about the necessary improvements required to make Latvia a more attractive place for remote workers, the respondents first point to the need to raise salaries (67.6%). From the state, they expect measures to ensure an adequate tax and social insurance system (43.2%), a simpler and easier to understand tax system (35.2%), and an adequate regulatory framework related to remote work (37.7%). From the employers they expect, besides higher salaries, improved trust and attitude toward workers which are especially crucial in cross-border employment (40.4%) (Figure 7).

Disorganized or complex legislation on tax issues related to remote work, both in Latvia, Europe and at the global level, is considered as the most important problem. Respondents draw attention to the ambiguities and disagreements that arise from the application of various regulatory frameworks in different countries, which, in their opinion, have not been sufficiently discussed either at the level of European or Latvian decision-making institutions. This creates confusion, misunderstandings, and even the threat of real sanctions, and can make it difficult and not appealing to work remotely across borders. Participants of the in-depth interviews suggested that the issue of social guarantees for those performing cross-border remote work should be addressed at the international level, and it is Latvia that could initiate that, at least at the EU level, social guarantees in the case of cross-border remote work are harmonized and explained in a comprehensible way to both employers and employees. At the moment in the EU and in the case of bilateral social security agreements there are coordination provisions that allow determining which country is responsible for specific social guarantees, and it is possible to receive the services of the social security system even if a person lives in a member state other than the one where he works or has worked, the system can be quite complex and unclear, and even more problems arise in case there are no bilateral agreements between counties.

“*These formal matters, which could make remote work difficult, social guarantees, should be emphasized more and discussed in a wider context. Because, in principle, not so much is said about it. [..] For Europe to facilitate it and not slow down [cross-border remote work]. There must be some kind of more favorable conditions for people to have clarity and also security.”* (RN11_2021)

A total of 28.5% think that securing a more stable and predictable economic environment would help, while 18.9% believe that tax breaks for remote workers could make the country more attractive for such employment. Currently, according to our interviewees, low competitiveness of the Latvian labor market and general economic problems lower the attractiveness of the business environment in Latvia and hinder recruitment of qualified labor, including specialists to perform remote work.

Some of the proposals refer to the insufficient readiness of companies in Latvia to this form of employment: 25.1% believe that remote work opportunities in Latvian companies are insufficient in general, and 29.2% state that the level of digitization of the employers should be improved.

For those who work remotely for Latvian companies or organizations, an opportunity to arrange various formalities Online is also critically important. Currently the majority of cross-border remote workers (61.8%) are generally satisfied with the availability of digital public services in Latvia, i.e., the possibility to resolve various issues and apply for services in public administration institutions online, however 18.5% are rather or completely dissatisfied with the availability of digital public services in Latvia. This can hinder attracting remote workers from abroad. An important prerequisite to being able to use the e-government services is an e-signature. This facilitates the circulation of documents in Latvia but unfortunately for various reasons is not available to all members of the diaspora and does not work the same way in other countries. Interviewees voiced the opinion that it would be very valuable if an e-signature were uniform in all EU member states: “*In Latvia, everything can be done with an e-signature because it is an important component, and it works here. It would be cool to have some kind of global solution like that*.” (RN8_2021)

## 4 Conclusions

### 4.1 The potential of cross-border remote work

This study points to the untapped potential of cross-border remote work, and confirms the need for support tools and solutions aimed at cross-border remote workers, with a view to their possible return (considering that the remote job can become the first step or a connection facilitating return).

According to previous research, approximately a third of all workplaces could be located at home, but in some industries such as IT, finance and insurance, professional, scientific, and technical services, and education, the number is much higher. Nevertheless, just 13% of workers in the EU currently perform their work remotely. According to our study, cross-border employment so far constitutes a small share of remote jobs even among diaspora (17%), which means that the potential of not just remote work in general, but cross-border remote work in particular, remains largely untapped. This is especially the case for the government sector.

A limitation of cross-border work is that in many cases only those positions that allow full-time or close to full-time remote work are suitable. The answers obtained in this study paint a promising picture for cross-border work, as most remote jobs are performed fully or close to fully remotely—and even in those that are not, more than half could be performed fully remotely. Moreover, at least sometimes alternating between countries is so easy that even partial remote work becomes suitable for cross-border employees. We can conclude that the vast majority of remote jobs are suited for cross-border employment, and the main limiting factors of this form of work are elsewhere.

Besides the untapped potential of cross-border work in general, our study reveals that even those members of the Latvian diaspora who can and do perform their duties remotely across borders, very rarely invest their knowledge in Latvian companies and institutions. This means that by improving the prerequisites for performing remote work, both in the private and the public sector, there are significant opportunities to improve the involvement of the diaspora in the Latvian labor market. The main reason why people are not so convinced about the competitiveness of Latvia's business environment in terms of attracting professionals living abroad to work remotely for companies or organizations in Latvia, are the comparatively low salaries. However, other issues such as the digital readiness of companies and the tax and social insurance system also require immediate attention in the context of the rise in cross-border remote work.

### 4.2 The policy perspective

Our analysis suggests that, beyond various guidelines, currently there is no good regulation for employing people remotely across borders: National policies and even the definition of remote work differ, and a lot depends on bilateral agreements of the involved countries. Most often, remote employees choose to work as self-employed, yet it does not provide them with the same sense of security and places the entire administrative burden directly on the employee's shoulders. In some cases, especially if several employees are hired from the same country, a labor hire service may be used, which in turn imposes additional administrative costs on the employer. Therefore, as our interviews of remote workers suggest, some companies in Latvia simply choose to ignore the fact that the worker resides abroad, use the regular contracts and the worker remains as tax resident in Latvia. It is less common for a company to enter employment contracts with non-residents who work remotely, as social benefit systems are poorly connected at the international level. This is particularly the case for countries beyond the EU borders that do not always have a bilateral taxation/social security agreement, but even within the EU, problems with access to social security and basic social services are common. In cross-border working arrangements, many questions and uncertainties arise for both parties, such as the application of the tax system and social security payments, which differ significantly between countries. The current complex system can be considered as far from ideal.

Changes in regulatory acts and the introduction of support measures aimed not only at improving the remote work environment, but also at the return migration of remote workers living in the diaspora, are in the national interest of countries such as Latvia. This is evidenced by the demographic and professional profile of remote workers, which directly corresponds to the sectors and areas in which there is a shortage in the Latvian labor market. As was concluded both during the secondary data analysis and in the survey, remote employees in the diaspora are significantly more likely to be employed in STEM industries, are more often leading specialists or specialists with higher education, are less rooted in their countries of residence, and are more willing to consider return than other members of the diaspora. Recognizing this potential, an increasing number of countries introduce digital nomad visas, e-residency, and tax exceptions for remote workers moving their tax residence there.

Our diaspora survey suggests that ensuring simple and favorable regulations and conditions may play a significant role in whether people will choose to engage in remote work in or from a certain country. For example, just 18% of Latvian remote workers live in the UK—a small number compared to the overall size of the Latvian diaspora there. Significantly more often remote workers from Latvia have chosen Eastern European countries, CIS countries, Georgia, Ukraine, Israel as their place of residence, and they are also more often found in southern European countries or in less diaspora-populated countries elsewhere in the world. This choice may be related to differences in the cost of living and a better climate, or to relatively more flexible working conditions and a more attractive tax regime. In any case, the perspectives of remote work will clearly depend on the ability of countries to ensure satisfactory cross-border work regulations.

### 4.3 The attitude of employers

While approximately a half of companies in Latvia employ workers in a remote mode, the practice of employing employees who live permanently abroad is currently extremely rare (4.4% of companies have remote workers). However, judging from the attitudes of employers, there is great potential in this form of work: 23.4% are open to having cross-border workers in the future. The reasons why most companies have a strongly negative attitude toward expanding their recruitment to people from abroad are related to perceptions of the difficulties that could arise in cross-border employment, especially difficulties for workers to separate private and work life, and frequent misunderstandings and confusion caused by remote employment, which they probably expect to be exaggerated by such a work arrangement, causing these employees to be less efficient. Many consider it necessary to meet in person at least now and then, which in cross-border employment is difficult.

The experience of companies employing cross-border workers indicates that there are objective difficulties that companies face in employing workers who live permanently outside the country, and that need to be addressed. The data also reveal that employing cross-border employees may exaggerate such issues as insufficient digital skills of employees, and the control and registration of their working hours. Being in another country can make providing the necessary support—as well as efficient control—difficult. Thus, cross-border remote work requires better and more flexible management.

Openness to cross-border employees also depends on whether the company is sufficiently savvy in digital technologies to solve any possible issues, as well as the number of employees working remotely from a specific country. Employing someone from abroad is a complex task: it involves understanding the taxation nuances and other legal cross-border regulations, and those companies where the number of remote workers is small might not find it worthwhile.

Respondents' answers also suggest that allowing employees to work from another country requires having sufficient trust in these workers being responsible and able to work effectively even in such conditions. Thus, a general lack of trust in the society may hinder the rise of cross-border work.

Interviews reveal that legal complexity and lack of clarity regarding taxation are some of the main problems preventing employers from hiring remote workers abroad, as well as difficulty in making sure that the workplace is equipped according to the regulations. Technical issues with the circulation of documents, data security, as well as interviewing and hiring cross-border workers are also elements of concern. As a result, only when employers have sufficient digital skills, favorable legal conditions, efficient management and trust in workers being able to work efficiently even in cross-border situation, would they consider this form of employment.

### 4.4 The attitude of employees

Our study reveals significant interest among remote workers in connecting their professional career or place of residence to Latvia, especially if the remote work environment is improved and organized. Despite difficulties (work outside the “standard” working hours, difficulties in separating private life from work, insufficient communication with colleagues, differences in time zone, etc.), of those who currently live and work remotely abroad, 40.1% would be ready to work remotely for a company or organization in Latvia (while living abroad), while many found this question difficult to answer, as it depends on various circumstances and conditions. The fact that cross-border workers are very satisfied with their job, as well as other aspects of their life, attests to the long-term potential of this form of work. With employers becoming more open to cross-border work, many more members of diaspora may use the opportunity to keep their employment abroad and return home. The potential to attract those living abroad to work in companies in Latvia is smaller, but still substantial, and it will depend on conscious state policy to make such a move more convenient.

Our study reveals that there are specific categories of people for whom cross-border work opportunities are very important and that specifically look for this kind of job: global nomads, people in transnational relationships, and those joining their spouse abroad with no knowledge of the local language. Thus, cross-border employment opens up significant opportunities to increase the total volume of the Latvian labor market with “digital labor” from abroad.

There are, however, several important challenges associated with this kind of work. Compared to those working remotely in their country of residence, cross-border remote work more often requires equipping oneself with the necessary technologies and resources, as the employers often fail to do so. Employees need to be able to arrange, both practically and legally, the possibility of working remotely across borders—as self-employed or in some other status. The work is generally more demanding in terms of organizing one's own work, integrating in the team, and efficiently meeting the deadlines. More often than other remote workers, cross-border employees have had to work while ill, which, as our data show, is likely related to the difficulties in arranging the formalities for sick leave in another country, and confusion as to if and where the person qualifies for healthcare services. Confusion with taxes and social benefits is another challenge. It is extremely concerning that, as the survey reveals, many cross-border workers living abroad but working in Latvia do not know where they can claim unemployment and maternity/parental benefits or think that they cannot get them anywhere. Respondent's answers about their tax residence implies confusion which has profound implications for where income should be reported and taxed. We can conclude that a large part of those working in Latvia but living abroad do not have access to the social support that is available to other residents of Latvia. For those who are self-employed, this status in itself comes with concerns regarding stability of income and social guarantees in case of unemployment or illness, and a lot of stress.

## 5 Discussion

### 5.1 What does the future hold?

While after the end of the COVID-19 pandemic some companies will cut down on remote work, at least to an extent, the tendencies are clear—this form of work is here to stay, and with increasing digitalization and sophistication of technologies its use in the future will grow. The pandemic served as a turning point, in Bourdieu's (1993) words, creating hysteresis disturbances between the field and habitus, where in the light of the rapid changes individuals had to adopt to different habits and seek different solutions than what they had been used to. Importantly, after hysteric changes, the system never goes back to where it was. Our survey data once again underline that the transition to remote work is a part of the long-term labor market transformation process and not a short-term shock adaptation mechanism caused by the pandemic.

The world after the COVID-19 pandemic is not as geographically bonded as before—work teams increasingly consist of people in different countries, all part of a “global network.” Closely knit, well-structured hierarchical groups give way to “networked individualism” (Castells, 2009) where employees increasingly need to organize their own work. These changes create new challenges, and necessitate adaptation both on the part of employees, as well as managers and employers.

The increasing technological possibilities—the use of various communication platforms, applications, modern work control and security systems—as well as the changing habitus of employees and employers in respect to remote employment, have made it much simpler and practically feasible to hire employees from other countries. The rise of cross-border remote work, as we argue in this paper, creates opportunities for countries suffering from emigration-induced brain drain. It makes it possible to attract qualified labor from abroad, including members of the diaspora who are often eager to contribute to their country of origin. It may also create more opportunities for those who wish to return, as they can now be with their loved ones while keeping their better-paying job abroad. However, benefiting from the opportunities provided by cross-border work requires proactive actions on the part of national and supra-national politicians, horizontal changes in regulatory acts, and an array of state support measures.

### 5.2 Recommendations and avenues for further work

As mentioned before, Latvia and other countries in Central and Eastern Europe have significant advantages that may allow them to become not only a regional, but also global leaders in remote work: a fast Internet connection; good transport connections with other European and Commonwealth of Independent States (CIS) countries; a wide range of digital services; relatively fast adoption of digital technologies in the private sector; and a number of other factors. Those working remotely are satisfied with their job and most would like to continue working remotely, thus, cross-border employment provides an opportunity for countries suffering from emigration to return their lost human capital in the form of “digital migration.” The answers of our respondents, however, reveal a belief that Latvia could do better in attracting cross-border workers: just 20% rate Latvia's business environment as good for attracting people living abroad to work remotely for companies or organizations in Latvia. The study clearly highlights several challenges that hinder these benefits from transforming into a permanent flow of “digital return migration.”

For the national and international authorities, it is necessary to work on adjustments to the labor market legislation and regulations, tax system and regulation, and social protection of remote workers engaged in cross-border employment. The issues to solve also include aspects linked to digitalization: improvements in public services and urban environment that would make remote work more convenient (e.g., high-speed Internet in rural areas, expanding the range of digital public services), training in digital skills and networking (digital nomad communities, networking between remote jobseekers and employers), as well as support for entrepreneurs in the form of grants or tax deductions for digitalization, innovation in remote work technologies, and setting up a remote workplace. For the country to fully take advantage of the new opportunities, campaigns could also be implemented, e.g., advertisements for remote work opportunities in the country, public campaigns of the country as a great place for remote workers. Finally, the ability of Latvian companies to attract cross-border workers from abroad will to a large extent also depend on the competitiveness of wages, and the stability and predictability of the economic environment in general.

In terms of legislation, at the national level it is necessary to define the notion of “cross-border remote work,” and the legal rights and obligations of these workers. Other recommendations include removing the barriers to cross-border remote employment such as reviewing the rules and requirements for the protection of intellectual property when remotely employing employees who are physically located in another country, incorporating into the labor market legislation the right for employees to request interviews to be held remotely, especially in public administration and in positions that are intended to be conducted remotely in whole or in part, and following the Irish example (Government of Ireland, 2019), to introduce a norm into the labor market regulatory acts, which provides employees in professions where the specifics of the job allow it, the right to request the opportunity to work remotely for a certain period of their working time. This would provide more options to those that for one reason or another are not ready to move to Latvia fully. Considering that currently one of the biggest obstacles to cross-border employment is the complex bureaucracy linked to this form of work, the authorities could evaluate the possibility of introducing a simplified tax status for cross-border remote workers who are employed abroad as their main job, and creating substantially simplified informational materials and educational events explaining practical steps on the application of taxes in the case of cross-border remote work. Finally, the introduction of e-residency for remote workers, similar to those available in Estonia, as well as, possibly, short-term tax deductions for those non-nationals who move their tax residence to the country should be considered. In regards to digital nomad visas, the imposed minimum salary requirement is problematic and should be significantly softened, as nowadays not only highly-skilled workers can have an opportunity to engage in remote employment. As our survey indicates, many of those who can and do work remotely (for example, those employed in education, creative industries, hospitality, or sales), do not fulfill this requirement.

At the international level it is necessary to review the existing international agreements on the avoidance of double taxation, evaluating the incorporated taxation mechanisms in relation to cross-border remote work. It is also necessary to include standardized conditions regarding cross-border remote work in international agreements with countries with which negotiations are ongoing and to evaluate the possibilities of amending agreements with countries with which non-standard practices have been established. The share of countries with which double-tax avoidance agreements have been concluded needs to be expanded, too.

In terms of social security, it is necessary to expand and facilitate the availability of social guarantees for remote workers, especially to those who are self-employed or have non-standard contracts. It is also crucial to simplify the transfer of social contributions from foreign countries, if a person decides to move. The availability of social guarantees for remote workers in foreign countries who make social contributions in another country, but do not have a declared place of residence in their country of residence needs to be reviewed, especially if the employment is in a country that does not have bilateral agreement with the country of residence. Considering the doubts and fears surrounding social guarantees for cross-border workers, it is necessary to significantly simplify the information on the availability of social guarantees in the case of cross-border remote work or, in case of Latvia, to increase awareness of an opportunity to voluntarily join the social insurance system.

It would also be useful to prepare informative materials and implement trainings on effective organization and registration of remote work, taxation, social guarantees and other issues for both remote work performers and employers, and to create an environment in which companies and labor market participants can exchange good practices in the organization of remote work, including developing samples of employment contracts for entrepreneurs suitable for remote work.

This study has outlined the opportunities and challenges related to cross-border remote employment for countries that suffer from emigration-induced human capital loss, yet more studies are needed that would go beyond descriptive analysis and explore in detail the possible legislative solutions that would make cross-border employment more attractive and feasible for both employers and employees. Moreover, the answers of remote workers suggest that there is an even higher potential to convince people to live in Latvia and work for a company abroad rather than the other way round. Thus, this is another option to explore in future studies.

## Data availability statement

The raw data supporting the conclusions of this article will be made available by the authors, without undue reservation upon request.

## Ethics statement

Ethical approval was not required for the studies involving humans because the study was a continuation of an earlier research project on Latvian diaspora conducted in 2019 that was extensively reviewed and approved by the University of Latvia Ethics Committee. At all stages, researchers acted in accordance with all national and EU data protection and ethical regulations, including GDPR. All research participants were provided with written description of their rights as respondents, information on data storage and protection etc. to the highest standards, and required written consent. The studies were conducted in accordance with the local legislation and institutional requirements.

## Author contributions

IM: Formal analysis, Methodology, Project administration, Visualization, Writing – original draft, Conceptualization, Investigation. IŠ: Conceptualization, Formal analysis, Methodology, Investigation, Writing – review & editing.
